# Angiogenic Gene Signature Derived from Subtype Specific Cell Models Segregate Proneural and Mesenchymal Glioblastoma

**DOI:** 10.3389/fonc.2017.00146

**Published:** 2017-07-11

**Authors:** Aman Sharma, Ajinkya Bendre, Abir Mondal, Dattatraya Muzumdar, Naina Goel, Anjali Shiras

**Affiliations:** ^1^National Centre for Cell Science (NCCS), SP Pune University Campus, Pune, India; ^2^ExoCan Healthcare Technologies Pvt Ltd, Venture Centre, NCL Innovation Park, Pune, India; ^3^Seth G.S. Medical College, KEM Hospital, Mumbai, India

**Keywords:** angiogenesis, intertumoral heterogeneity, angiopoietins, glioma stem cells, biomarker

## Abstract

Intertumoral molecular heterogeneity in glioblastoma identifies four major subtypes based on expression of molecular markers. Among them, the two clinically interrelated subtypes, proneural and mesenchymal, are the most aggressive with proneural liable for conversion to mesenchymal upon therapy. Using two patient-derived novel primary cell culture models (MTA10 and KW10), we developed a minimal but unique four-gene signature comprising genes vascular endothelial growth factor A (VEGF-A), vascular endothelial growth factor B (VEGF-B) and angiopoietin 1 (ANG1), angiopoietin 2 (ANG2) that effectively segregated the proneural (MTA10) and mesenchymal (KW10) glioblastoma subtypes. The cell culture preclassified as mesenchymal showed elevated expression of genes VEGF-A, VEGF-B and ANG1, ANG2 as compared to the other cell culture model that mimicked the proneural subtype. The differentially expressed genes in these two cell culture models were confirmed by us using TCGA and Verhaak databases and we refer to it as a minimal multigene signature (MMS). We validated this MMS on human glioblastoma tissue sections with the use of immunohistochemistry on preclassified (YKL-40 high or mesenchymal glioblastoma and OLIG2 high or proneural glioblastoma) tumor samples (*n* = 30). MMS segregated mesenchymal and proneural subtypes with 83% efficiency using a simple histopathology scoring approach (*p* = 0.008 for ANG2 and *p* = 0.01 for ANG1). Furthermore, MMS expression negatively correlated with patient survival. Importantly, MMS staining demonstrated spatiotemporal heterogeneity within each subclass, adding further complexity to subtype identification in glioblastoma. In conclusion, we report a novel and simple sequencing-independent histopathology-based biomarker signature comprising genes VEGF-A, VEGF-B and ANG1, ANG2 for subtyping of proneural and mesenchymal glioblastoma.

## Introduction

Gliomas account for ~30% of all brain and central nervous system tumors and 80% of all malignant brain tumors. The highly malignant grade IV glioma referred to as glioblastoma is associated with poor patient prognosis and shows median patient survival of only ~12–14 months (1, 2). Glioblastoma is considered to be driven by a subpopulation of brain tumor-initiating cells, and presence of these cells within the tumor contributes toward cellular plasticity and heterogeneity (3, 4). The regulatory landscape of glioblastoma has led to its categorization into four major molecular subtypes: neural, classical, proneural, and mesenchymal (5–7). Each of these subtypes harbor unique genomic and epigenomic regulatory features and are clinically independent and manifest various prognostic significances.

There is a significant level of intertumoral as well as intratumoral heterogeneity within the four glioblastoma subtypes and this is a potential impediment of patient outcome during therapy (5, 8). Spatiotemporal heterogeneity is also manifested at a single-cell level and causes coexistence of multiple molecular subtypes within a single glioblastoma tumor often yielding chimeric glioblastoma cell clones (2, 8). The possibility of subtype switch in glioblastoma induced by chemotherapy and the high levels of intertumoral heterogeneity necessitates accurate identification of tumor subtypes in glioblastoma (9, 10). The various data sets generated by TCGA analyzed through genetic, gene expression, and DNA methylation signatures have led to the identification of divergent glioma subtypes elucidated on the basis of the status of the IDH1 gene, codeletion of chromosome arm 1p/19q, and TERT promoter status (11).

Among the glioblastoma subtypes, it is proposed that upon recurrence, glioblastoma tumors become mesenchymal whereas the tumors with the proneural signature show worst prognosis upon treatment (7, 12). It has been reported that glioblastoma may be derived from a common proneural-like precursor (13). Here, we report development of a novel and robust immunohistopathological-based minimal multigene signature (MMS) for effective segregation of the mesenchymal and proneural glioblastoma subtypes. This segregation signature was developed by us using prototype, proneural, and mesenchymal subtype-specific glioblastoma long-term cell cultures and comprises four genes: angiopoietin1 (ANG1), angiopoietin-2 (ANG2) and vascular endothelial growth factor A (VEGF-A) and vascular endothelial growth factor B (VEGF-B). These genes play critical roles in angiogenesis and we discovered that expression of the genes was an effective subtype classifier for the two glioblastoma subtypes. This four-gene signature was very effective in proneural and mesenchymal segregation on the basis of preclassified glioblastoma histology, signifying its use in clinical practice. Importantly, we were able to effectively capture tumor heterogeneity both at the tumor and the single-cell level and positively correlate it with cellular diversity estimation and patient survival outcome. We propose that the use of this gene signature classifier under clinical settings has significant bearing on therapy regime and patient outcome.

## Materials and Methods

### Generation of Primary Glioblastoma Cultures

Two long-term primary cell cultures were established from surgically excised tumor tissues using a previously described method (3). Single cells obtained by digestion with collagenase type IV (1 mg/ml; Gibco) were plated using DMEM/H12 medium containing 10% fetal bovine serum and 1× P&S antibiotic solution. Early passage cultures were used in all experiments. This study was approved by the Institutional Ethics Committee of Seth GS Medical College & KEM Hospital, Mumbai (India) and NCCS, Pune (India).

### PKH Dye Dilution Assay

Single-cell suspensions of KW10 and MTA10 cells were labeled with PKH-26 dye (Sigma) method as described by Givan et al. (14). Briefly, 2 × 10^7^ cells were labeled with 4 µl of PKH-26 dye and analyzed using a 567 nm laser on a BD FACS Aria cell sorter and FACS DIVA software (BD Biosciences).

### Immunofluorescence and Western Blotting

Subconfluent cultures were fixed using 4% paraformaldehyde at room temperature (RT) for 10 min, followed by permeabilization with 0.01% Triton ×100 for 4 min. Cells were stained with primary antibodies—YKL-40 (sc-393590), VEGF-A (sc-152), and VEGF-B (sc-1876) from Santa Cruz Biotech, Olig 2 (ab42453), MAP2 (ab11267), ANG1 (ab8451), and ANG2 (ab8452) from Abcam, GFAP (mab 360) from Millipore, and nestin (N5413-R) from Sigma, for 2 h at RT. Subsequently, cells were stained with Alexa Fluor-conjugated secondary antibodies (Invitrogen). For Western blotting, cell monolayers were washed thrice with 1× PBS and harvested using trypsin. Cell pellets were lysed in MPER containing 1× protease inhibitor cocktail (Thermo Scientific). A total of 40 µg of cell lysate was loaded in each experiment, and electrophoresed samples were transferred onto PVDF membrane (Pall Life Science). Membranes were probed using respective primary antibodies (CD44, HPA005787, dilution 1:1,000, Sigma; α-tubulin, T9026, dilution 1:5,000, Sigma; and VEGF-A, VEGF-B, ANG1, and ANG2 at 1:1000 dilution) overnight. Membranes were washed thrice in 1× PBST and probed with secondary antibody (antimouse HRP, 616520, dilution 1:5,000; antigoat HRP, 611620, dilution 1:1,000; and antirabbit HRP, 656120, dilution 1:1,000; Invitrogen) for 2 h at RT. Blots were developed using ECL substrate (Thermo Scientific).

### Scratch Assay

For scratch assays, cells were seeded into six-well plates in DMEMF12 culture medium and allowed to grow for 48–72 h until confluency was reached. Cells were washed with 1× PBS and a scratch was made using a 10 µl tip at the center of the well. Monolayers were imaged at the indicated time using a light microscope at ×100 magnification. Scratch healing was quantified using IMAGEJ software, and data were analyzed using Graphpad Prism 6 software.

### Neurosphere Formation Assay

Assay was performed by seeding single-cell suspensions into 96-well plates at a density of 100 cells per well in medium. Assays were performed over a period of 7 days.

### RT PCR and qRT-PCR Analyses

Total RNA was isolated from cells using Trizol Reagent (Invitrogen), and cDNA was synthesized using High Capacity cDNA Reverse Transcription Kit (Applied Biosystems). Expression of mesenchymal, proneural, and stemness genes was analyzed using both RT-PCR as well as qRT-PCR and gene-specific primers (Table S1 in Supplementary Material).

### *In Vivo* Tumorigenicity and Survival Analysis

2 × 10^5^ cells were injected orthotopically into brain of SCID mice. Brain tissue was harvested after neurological signs of cachexia, disturbed orientation, etc. H&E staining was performed to locate tumor regions within the brain parenchyma. For survival analyses, similar numbers of KW10 and MTA10 cells were injected orthotopically into brain of SCID mice, and the mice were monitored for their survival each day. Animal experiments were performed as per Institutional Animal Ethics Committee guidelines of NCCS, Pune, India.

### Immunohistochemistry (IHC)

Immunohistochemistry was performed on 5 μm-thick formalin-fixed and paraffinized sections of human glioblastoma tumor tissues. Sections were deparaffinized in xylene and dehydrated in alcohol gradient followed by blocking in 5% BSA in PBS. Next, sections were stained with primary antibodies: YKL-40 (sc-393590), VEGF-A (sc-152), and VEGF-B (sc-1876) from Santa Cruz Biotech, Olig 2 (ab42453), ANG1 (ab8451), and ANG2 (ab8452) from Abcam, followed by staining with appropriate Alexa Fluor-labeled species-specific secondary antibodies (Invitrogen).

### Histochemical Evaluation of MMS Expression

Five random fields (×63) for each mesenchymal or proneural glioblastoma tumor (each *n* = 10) were selected for analysis. Expression intensities were evaluated independently by two researchers. IHC scoring was performed as described earlier (15, 16). Expression intensities were preclassified into negative (−), weak (+), medium (++), and strong (+++) by calculating normalized values in ImageJ software. Cumulative IHC scores were calculated by a scoring scheme as (− = 0, + = 1, ++ = 2, and +++ = 3) and using formula (grade of intensity × number of samples)/total number of samples. Additionally, percentages of cells that were both weakly and strongly positive were evaluated. Finally, percentage positivity was calculated as number of weak (or strong) positive cells/total number of cells in a given field ×100.

### Single-Cell Heterogeneity Profiling

Tumor cells in glioblastoma tissues (1,000 cells/glioblastoma, *n* = 10 each glioblastoma subgroup) were selected in five random low power fields (×63). Tumor cells costained with YKL-40 and respective MMS glioblastoma genes were classified as double positive (DP), double negative (DN), and single positive or positive for respective MMS glioblastoma gene (SP). Individual tumor cells were color coded and plotted with a score criterion (DP = 1, SP = 0.5, DN = 0). Percentage positivity in each category was calculated using the formula: number of DP (or SP or DN) cells/total number of cells in given field ×100, and the same data were used for coexpression analysis.

### Patient Survival Analysis

Overall survival was calculated by Kaplan–Meier analysis using normalized RNA-Seq and microarray data from the TCGA database and by Verhaak et al. (5, 6). We classified patients into short-term survivor (STS) and long-term survivor (LTS) groups (17). Briefly, patients representing less than 25% of maximal survival were denoted as STS, whereas patients with more than 75% of maximal survival were assigned as LTS. Survival plots were analyzed using Graph Pad Prism software.

### Statistical Analysis

All statistical correlations were calculated using GraphPad Prism 5 software using unpaired *t*-test. Bars in all figures represent mean ± SEM. One-way ANOVA was used for stained area measurements after histochemistry. Pearson correlation was computed using an IHC scoring matrix generated from a total of 20 glioblastoma tissues (*n* = 10 for each subtype).

## Results

### Glioblastoma-Derived Cell Cultures Show Different Proliferation Potentials

**Figure 1:**
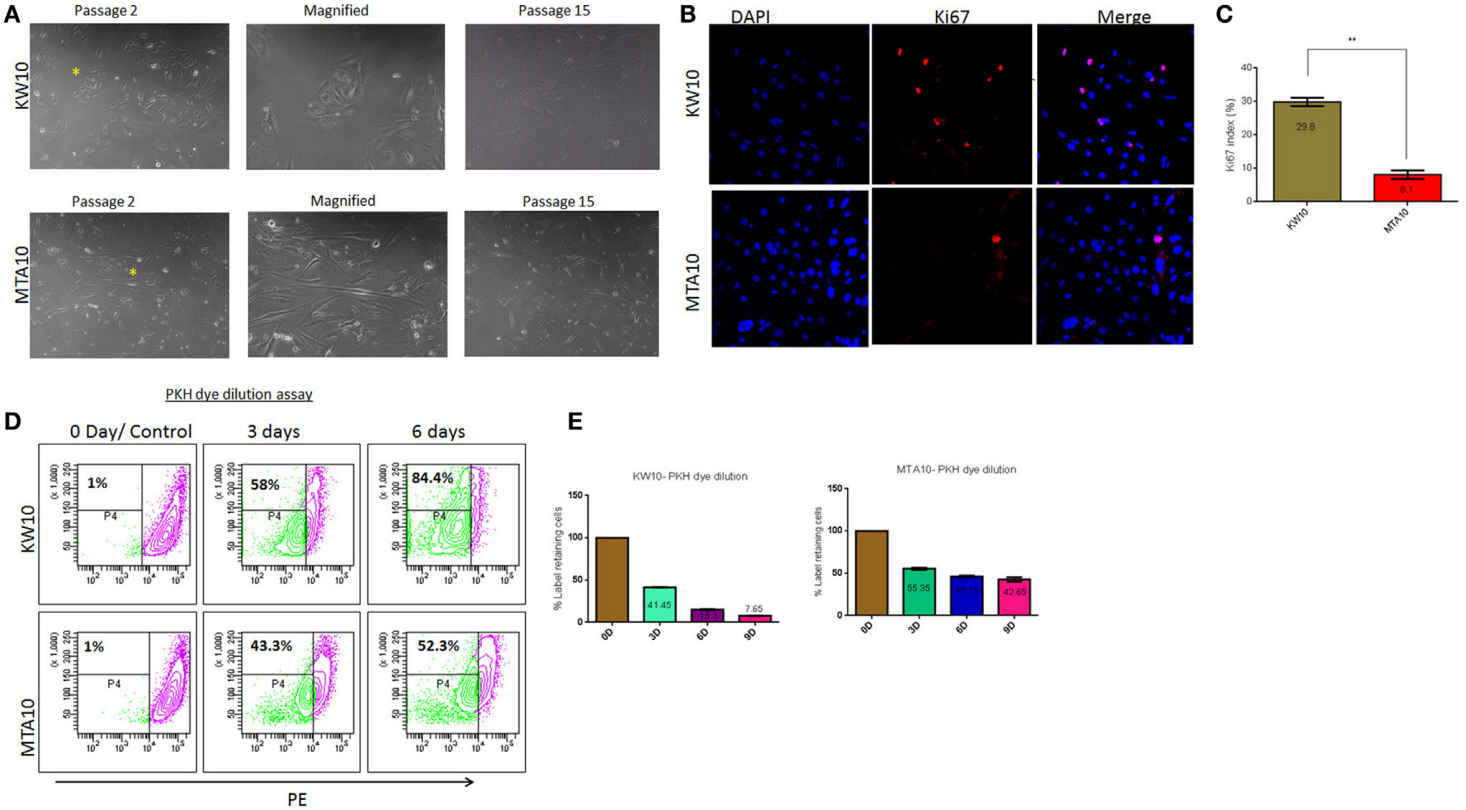
Establishment and phenotypic characterization of primary glioblastoma cell cultures—KW10 and MTA10. **(A)** Phase contrast micrographs of glioblastoma cells and at passage 15. **(B)** Ki67 staining. **(C)** Quantification of Ki67 expression (*n* = 3; **p < 0.001). **(D)** Quantification of cycling kinetics in a PKH dye dilution assay up to 6 days using flow cytometry. **(E)** Dye dilution quantified as mean ± SEM (*n* = 3; **p < 0.001).

Previously, we reported the development of model systems to study tumor progression in glioblastoma (3, 18). Here, we generated two distinct long-term cultures KW10 and MTA10 from glioblastoma tumors. KW10 cultures consisted of small, flattened cells that were morphologically uniform, whereas MTA10 cultures consisted of elongated cells that appeared predominantly neuronal (Figure 1A). Heterogeneous cancer cells in primary culture compete for nutrients. Often, only cell clones with growth advantages are selected and expanded in stable cultures (3, 19). We propagated both the cultures for >15 passages and found no significant changes in their growth or morphology over time (Figure 1A). Next, Ki67 staining showed a 3.7-fold higher positivity in KW10 cultures over MTA10 cultures (Figures 1B,C). Furthermore, the PKH dye dilution assay showed that over a period of 3 days, 58% cells were dividing in KW10 cultures as compared to only 43% cells in MTA10 cultures (Figure 1D). Moreover, analysis of cells over an extended period of time showed that >84% KW10 cells proliferated in contrast to only 43% cells of MTA10 culture. Strikingly, MTA10 cells retained PKH dye even after 9 days in culture (7.7 vs 42.7%), confirming its slow proliferative potential (Figure 1E). Moreover, cell cycle analyses showed significantly higher percentages of KW10 cells in S phase (Figure S1 in Supplementary Material). The differences in Ki67 positivity as well as the PKH dye dilution assay indicate that KW10 cultures represent the mesenchymal subtype and were highly proliferative as compared to MTA10 cells that showed the proneural subtype and were moderately proliferative.

### KW10 Cells Represent the Mesenchymal Subtype and MTA10 Cells the Proneural Subtype

**Figure 2:**
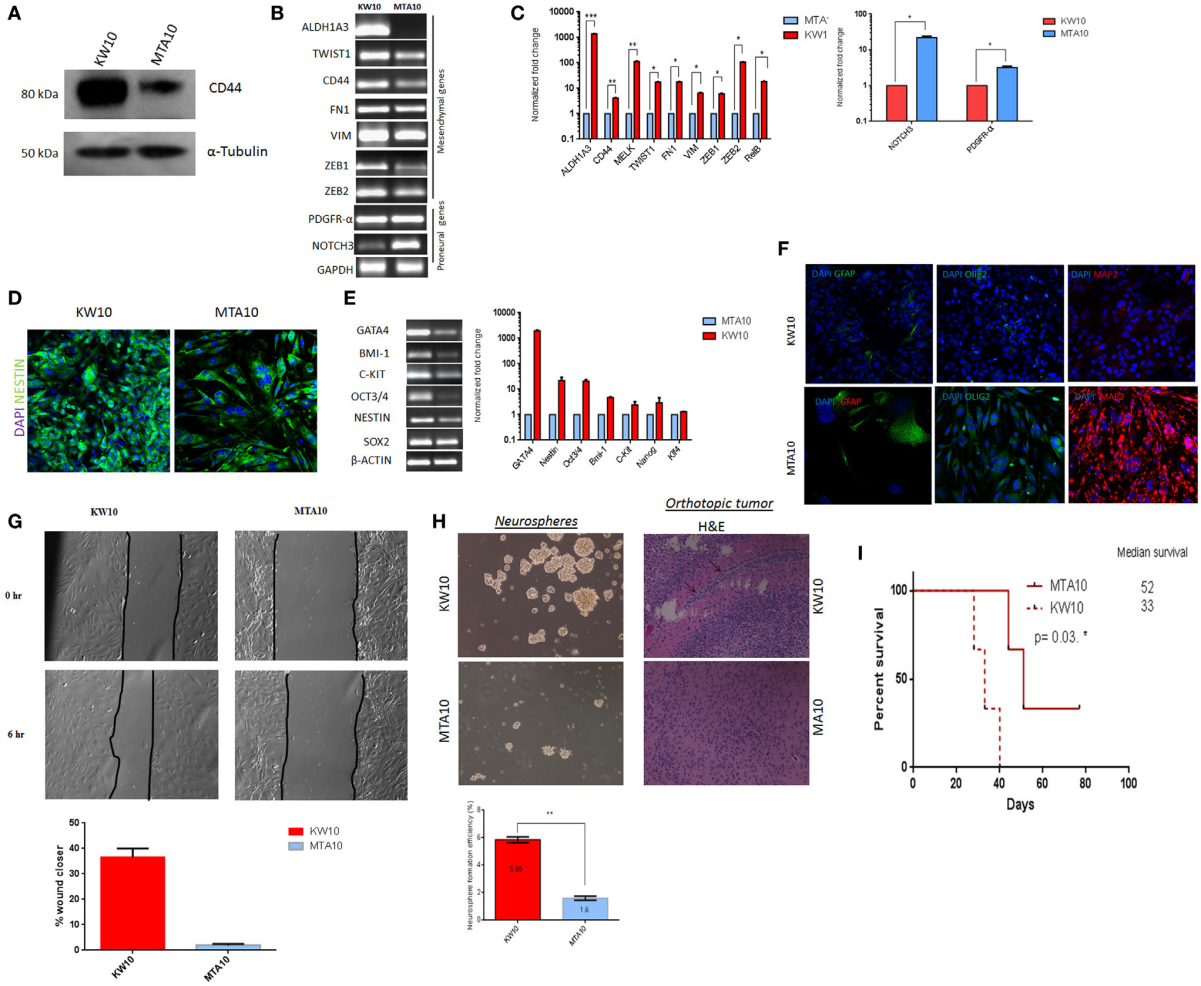
KW10 and MTA10 cell cultures represent mesenchymal and proneural glioblastoma subtypes. **(A)** Western blotting of the mesenchymal-specific marker CD44. **(B)** Semiquantitative RT-PCR for mesenchymal and proneural genes; GAPDH served as internal loading control. **(C)** qRT-PCR for known mesenchymal and proneural specific transcripts. Values represent normalized fold changes ± SEM of MTA10 and KW10 cell cultures, *p < 0.01. **(D)** Immunofluorescence detection of nestin expression. **(E)** RT-PCR analysis of stemness markers and quantification of stemness gene expression. Normalized fold change is represented as compared to MTA10 cells (mean ± SEM), *n* = 3. β-Actin served as internal control. PCR was performed in 7500 Fast real-time PCR system (Applied Biosystems) using SYBR Green Master Mix (Applied Biosystems). Data were normalized using the ΔCt method, and fold change was calculated using the 2^−ΔΔCt^ method. **(F)** Immunofluorescence staining of neural differentiation markers. **(G)** Scratch assay and quantification. **(H)** Neurosphere formation assay and its quantification. Data are represented as mean ± SEM, **p < 0.001, *n* = 3. Representative H&E staining of orthotopically implanted KW10 and MTA10 cells in mouse brain tumor tissue regions. Arrows show infiltrating cells within the tumor xenograft. **(I)** Survival analysis of KW10 and MTA10-injected SCID mice; data indicate that mice injected with KW10 cells show reduced survival (33 days) as compared to mice injected with MTA10 cells (52 days) (*n* = 3; *p = 0.03).

Morphological heterogeneity of cancer cells is often used in histopathology for prognostication (20, 21). To determine whether KW10 and MTA10 cell cultures represented distinct glioblastoma subtypes, we performed immunoblotting of the mesenchymal marker CD44 in both cultures (22). KW10 cells showed upregulated CD44 protein expression as compared to MTA10 cells, which indicates its mesenchymal origin (Figure 2A). Next, RT-PCR analysis of known proneural/mesenchymal subtype-specific genes revealed that KW10 cells expressed mesenchymal genes such as ALDH1A3, TWIST1, CD44, fibronectin (FN1), vimentin (VIM), ZEB1, and ZEB2, whereas MTA10 cells showed prominent expression of proneural transcripts such as PDGFR-α and NOTCH3 (Figure 2B). qRT-PCR showed significant upregulation of mesenchymal glioblastoma markers (>100-fold ZEB2; >10-fold TWIST1; FN1, <10-fold VIM; ZEB1; *p < 0.01) in KW10 cells as compared to MTA10 cells (Figure 2C). Upregulation of proneural transcripts (>10-fold NOTCH3; >3-fold PDGFR-α; *p < 0.01) was detected in MTA10 cells. A strong immunopositivity for the intermediate filament protein nestin was observed in KW10 cells (Figure 2D). Similarly, KW10 cells showed elevated expression of stemness genes like GATA4, OCT-3/4, and BMI-1 (Figure 2E). The qRT-PCR analyses showed significant upregulation of stemness genes in KW10 cells. Low expression of differentiation markers GFAP, Olig2, and MAP2 in KW10 cells indicated their poorly differentiated nature (Figure 2F). To evaluate the migration potential of both cell cultures, the scratch assay was performed. Data show higher wound closing potential in KW10 (37.8%) as compared to MTA10 (1.7%) (Figure 2G). In line, mesenchymal glioblastoma cells show expression of stem cell markers and are highly undifferentiated (9, 23). Conversely, MTA10 cells showed a differentiated phenotype and demonstrated stronger expression of neural lineage markers GFAP, Olig2, and MAP2 by confocal microscopy. Additionally, the neurosphere formation assay showed that KW10 cells possessed a higher capacity to form neurospheres as compared to MTA10 cells (*p* < 0.001, *n* = 3) (Figure 2H). In an orthotropic xenograft assay, KW10 cells formed highly infiltrative, aggressive tumors indicating the mesenchymal glioblastoma phenotype, as opposed to circumscribed tumors formed by proneural MTA10 cells (Figure 2H). Next, our survival assay demonstrated that mice injected with KW10 cells showed a median survival of 33 days as compared to mice injected with MTA10 cells that showed a median survival of 52 days (*p* = 0.03) (Figure 2I). The expression analyses as well as functional and survival studies for both cell types distinctly showed that they belonged to two independent subtypes with KW10 categorized by us as mesenchymal and MTA10 as proneural.

### Mesenchymal Subtype KW10 Cells Express Higher Levels of ANG1, ANG2 and VEGF-A, VEGF-B

**Figure 3:**
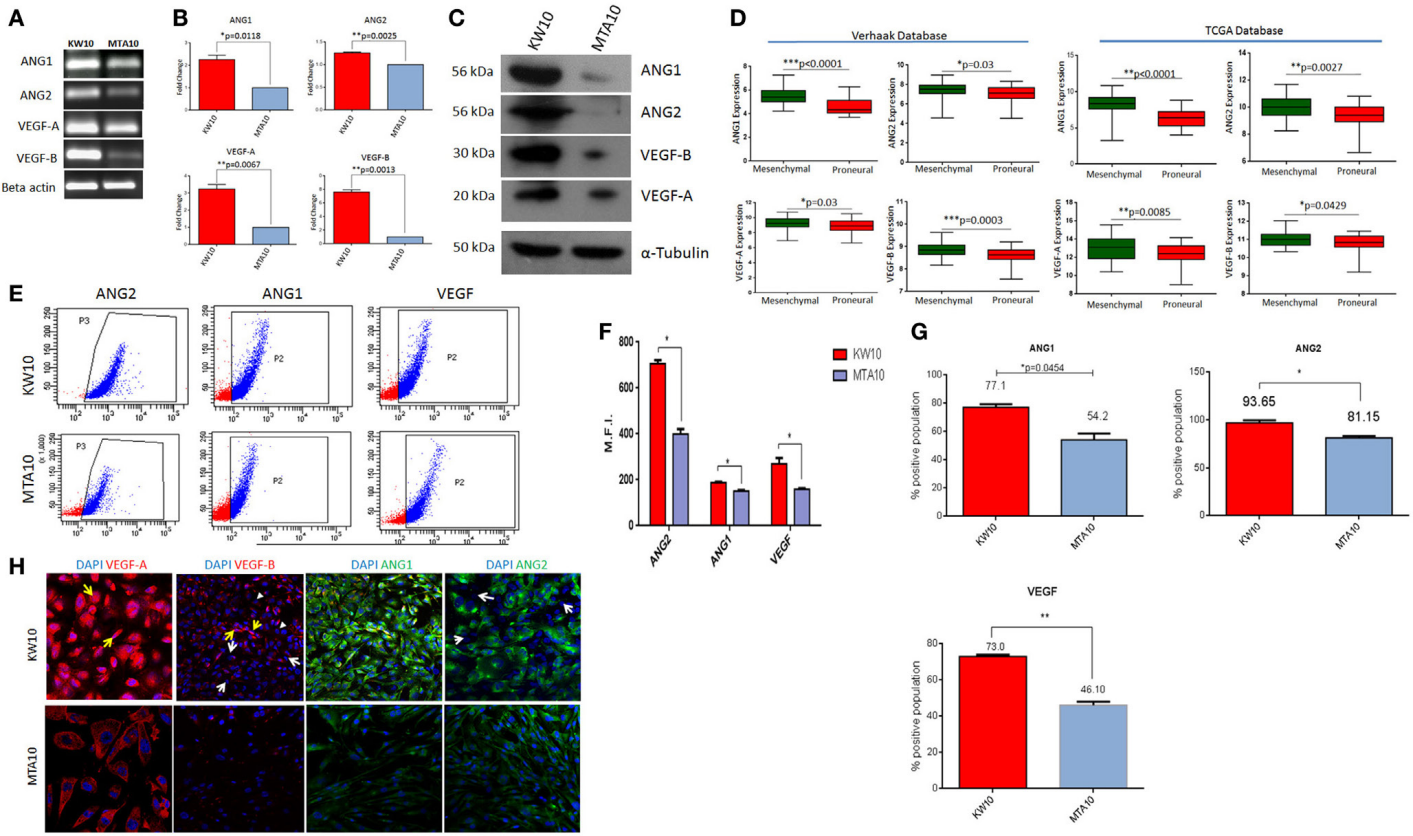
A minimal multigene signature (MMS) correlates with molecular subtypes in glioblastoma cultures. **(A)** RT-PCR analyses of the expression of angiopoietin 1 (ANG1), angiopoietin 2 (ANG2) and vascular endothelial growth factor A (VEGF-A), vascular endothelial growth factor B (VEGF-B). **(B)** qRT-PCR quantification of MMS glioblastoma. Normalized fold change is expressed in MTA10 cultures and is presented as mean ± SEM (*n* = 3). **(C)** Western blotting of angiogenesis-specific proteins in both cultures. **(D)** Validation of MMS glioblastoma in two independent glioblastoma data sets [TCGA, Verhaak (5, 6)]. **(E)** Scatter plots for ANG1, ANG2, and VEGF expression using flow cytometry and **(F)** quantification of mean fluorescence intensities (MFI) (*n* = 3). **(G)** Bar graph depicting percentages of positive cells in each gene belonging to MMS (*n* = 3 and data represented as mean ± SEM values; *p < 0.01). **(H)** Confocal imaging of immunostaining of MMS gene expression; arrows indicate cells showing differential expression of these proteins (white arrows, expression lacking cells; arrow heads, weakly expressing cells; and yellow arrows, highly expressing cells). Nuclear staining was performed using DAPI. Magnification ×63.

Mesenchymal glioblastoma tumors are hypoxic and marked by highly migratory cancer cells (24–26). To understand whether mesenchymal glioblastoma (KW10) cells express angiogenic pathway modulators, we determined expression of antagonistic pairs of angiopoietins (ANG1 and ANG2) and VEGF isoforms (VEGF-A and VEGF-B) in proneural (MTA10) and mesenchymal (KW10) cells. KW10 cells expressed ANG1, ANG2, VEGF-A, and VEGF-B transcripts at higher levels than MTA10 cells (Figure 3A). qRT-PCR showed that all four genes were significantly upregulated in KW10 cell cultures (ANG1, *p < 0.018; ANG2, **p < 0.0025; VEGF-A, **p < 0.0067; and VEGF-B, **p < 0.0013) as compared to MTA10 cells (Figure 3B). Coordinated interplay of ANG and VEGF isoforms regulates tumor angiogenesis (27, 28). Importantly, glioblastoma cells at invasion fronts express elevated ANG2 levels (29). To evaluate expression pattern of the four angiogenesis genes in both cell cultures, we performed Western blot analysis which showed markedly enhanced protein levels of angiopoietins and VEGF isoforms in KW10 and MTA10 cells (Figure 3C). Furthermore, analysis of two large glioblastoma data sets (5, 6) substantiated our findings and revealed prominent overexpression of ANG and VEGF isoforms in mesenchymal glioblastomas (Verhaak data set: ANG1, ****p < 0.0001; ANG2, *p < 0.03; VEGF-A, *p < 0.03; VEGF-B, ***p < 0.0003, and *TCGA* data set: ANG1, ****p < 0.0001; ANGPT2, **p < 0.0027; VEGF-A, **p < 0.0085; VEGF-B, *p < 0.0429) (Figure 3D). Similarly, flow cytometric analysis showed higher mean fluorescence intensity for ANG and VEGF proteins (Figures 3E,F) (ANG2, *p < 0.0074; ANG1, *p < 0.0132; and VEGF, *p < 0.046) as well as higher percentages of positive cells in KW10 cell cultures (ANG1, 77.1 vs 54.2, *p* < 0.045; ANG2, 93.65 vs 81.15, *p* < 0.0426; and VEGF, 73.0 vs 46.10, **p < 0.0063) (Figure 3G). Next, immunostaining for ANG1, ANG2, and VEGF-A, VEGF-B demonstrated expression heterogeneity between these two cells (Figure 3H). A strong nuclear expression of VEGF-A was observed in KW10 cells (Figure 3H, yellow arrow). Similarly, differential staining of VEGF-B in multiple cells (yellow arrows, high expression; white arrows, absence of expression; and arrow head, weak expression) was observed in KW10 cells. In contrast, MTA10 cells showed lower expression of VEGF-A and VEGF-B with more cytoplasmic staining (Figure 3H). Furthermore, heterogeneous ANG2 expression was evident in KW10 cells and ANG2-negative cells coexisted with ANG2-positive cells (white arrow), and MTA10 cells showed uniform but weaker staining of both proteins (Figure 3H). It is known that VEGF-B and ANG1 are both inhibitors of angiogenesis and are expressed during vessel stabilization (30, 31). Similarly, VEGF-A and ANG2 are pro-angiogenic modulators (32). In conclusion, our data indicate that an MMS consisting of four antagonistic angiogenesis ligand pairs (VEGF-A, VEGF-B and ANG1, ANG2) can successfully demarcate proneural and mesenchymal subtype cells in glioblastoma.

### YKL-40-Stained and OLIG2-Stained Regions As Potential Subtype Predictors

**Figure 4:**
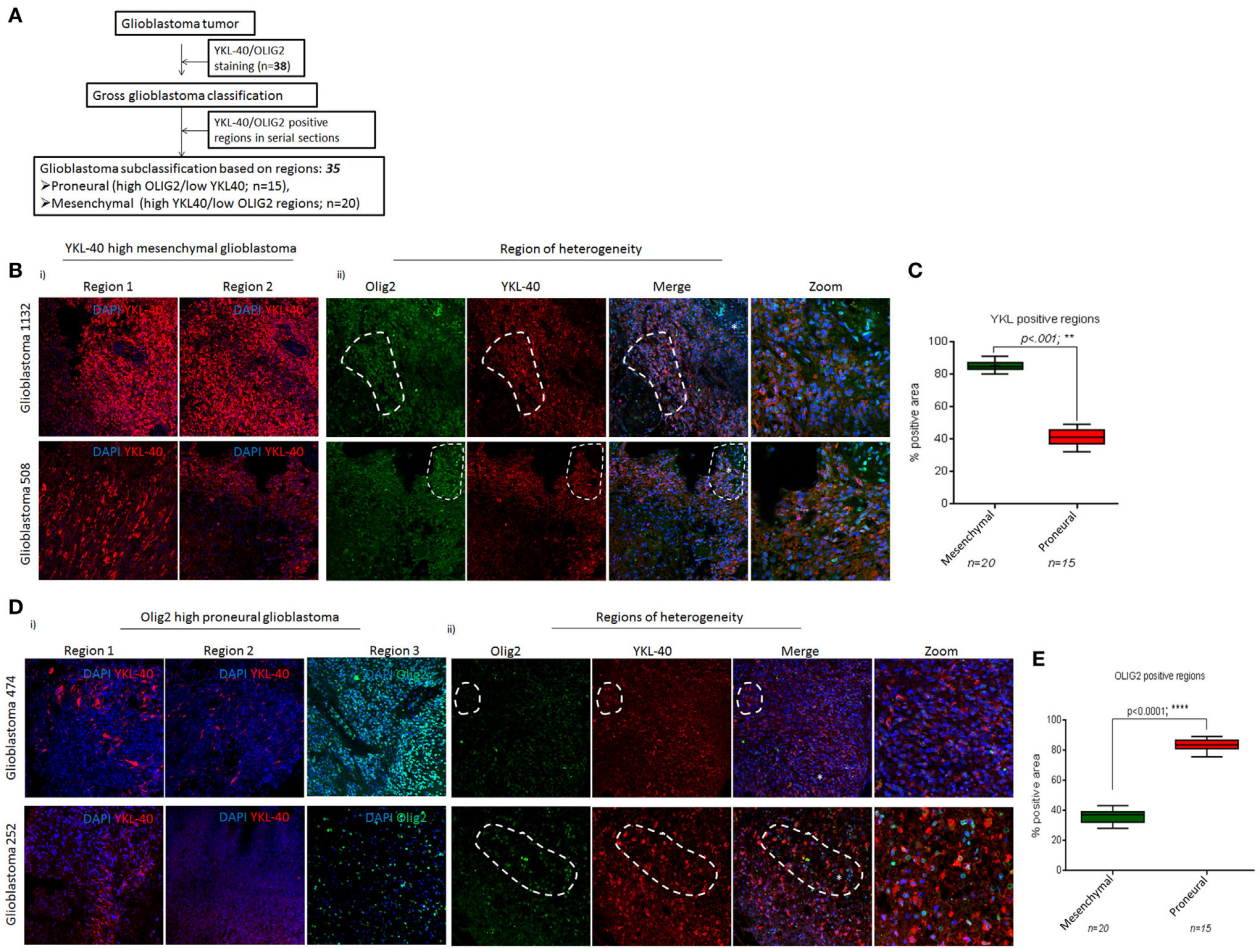
YKL-40 and OLIG2 positivity delineates mesenchymal and proneural glioblastoma subtypes. **(A)** Strategy for subtyping of glioblastoma tumors. **(B)** Immunohistochemistry shows bright YKL-40-positivity regions (i) and regions-of-heterogeneity (ROH) showing coexpression of both YKL-40 and OLIG2 in tumor cells (ii). **(C)** Semiquantitative analysis of YKL-40 immunostained areas for glioblastoma subclass segregation. Percent-positive areas are calculated from data shown in B and are plotted as mean ± SEM from glioblastoma samples (*n* = 35), **p < 0.001. **(D)** YKL-40 and OLIG2 immunostaining in glioblastoma tumors (i), ROH as areas with cancer cells coexpressing both YKL-40 and OLIG2 proteins (marked areas) (ii). **(E)** Semiquantitative analysis of OLIG2 immunostained areas for glioblastoma segregation. Percent-positive areas are calculated from **(D)**. Data are plotted as mean ± SEM from glioblastoma samples (*n* = 35), ****p < 0.0001. Magnification ×63. Sections were mounted in antifade mounting medium and acquired on a LSM 510, confocal microscope (Carl Zeiss AG).

Spatiotemporal heterogeneity in glioblastoma occurs due to coexistence of multiple subtype cell lineages and confounds molecular tumor pathology for diagnosis (2, 33, 34). In an attempt to classify glioblastoma tumors into proneural and mesenchymal subtypes, we performed IHC on 38 glioblastoma samples with markers such as OLIG2, PDGFR-α for the proneural and CD44, YKL-40 for the mesenchymal subtype. The overlapping of PDGFR-α and CD44 expression in serial sections hindered use of these molecules for subtype segregation. Hence, we focused on two other markers YKL-40 and OLIG2 for proneural and mesenchymal subtypes segregation using the strategy shown in Figure 4A. Glioblastoma tissue sections stained for YKL-40 and OLIG2 were screened in lower power fields (20×; 5 regions/sections in a minimum of 2–3 serial sections) and scored for YKL-40- and OLIG2-positive areas. Tumors with elevated YKL-40 expression with major regions being positive for this marker were classified as mesenchymal glioblastomas (85.2 vs 41.3; **p < 0.001, *n* = 20) (Figures 4Bi,C). Studies have pointed at the existence of hybrid cancer cells that coexpress two subtype-specific markers within a tumor (22, 33). Similarly, we found large areas in tumor sections coexpressing proneural and mesenchymal subtype markers OLIG2 and YKL-40 (Figure 4Bii, marked area). Therefore, to facilitate glioblastoma segregation into the two subtypes, we termed these areas as “regions-of-heterogeneity” (ROH) and excluded these from scoring. Conversely, tumors with weak YKL-40 positivity and strong OLIG2 sensitivity were categorized as proneural glioblastoma (*n* = 15) (Figure 4Di). We also observed ROH in proneural tumors, where both OLIG2 and YKL-40 proteins were coexpressed (Figure 4Dii, marked area). Proneural glioblastoma tumors contained more OLIG2-positive regions as expected (36.0 vs 83.4, ****p < 0.0001) (Figure 4E). Hence, by employing a simple two-step approach, we successfully preclassified a total of 35 (out of 38 tumors; 92%) newly diagnosed glioblastoma tumors into proneural and mesenchymal subtypes.

### MMS Glioblastoma As a Novel Proneural Mesenchymal Glioblastoma Classifier

**Figure 5:**
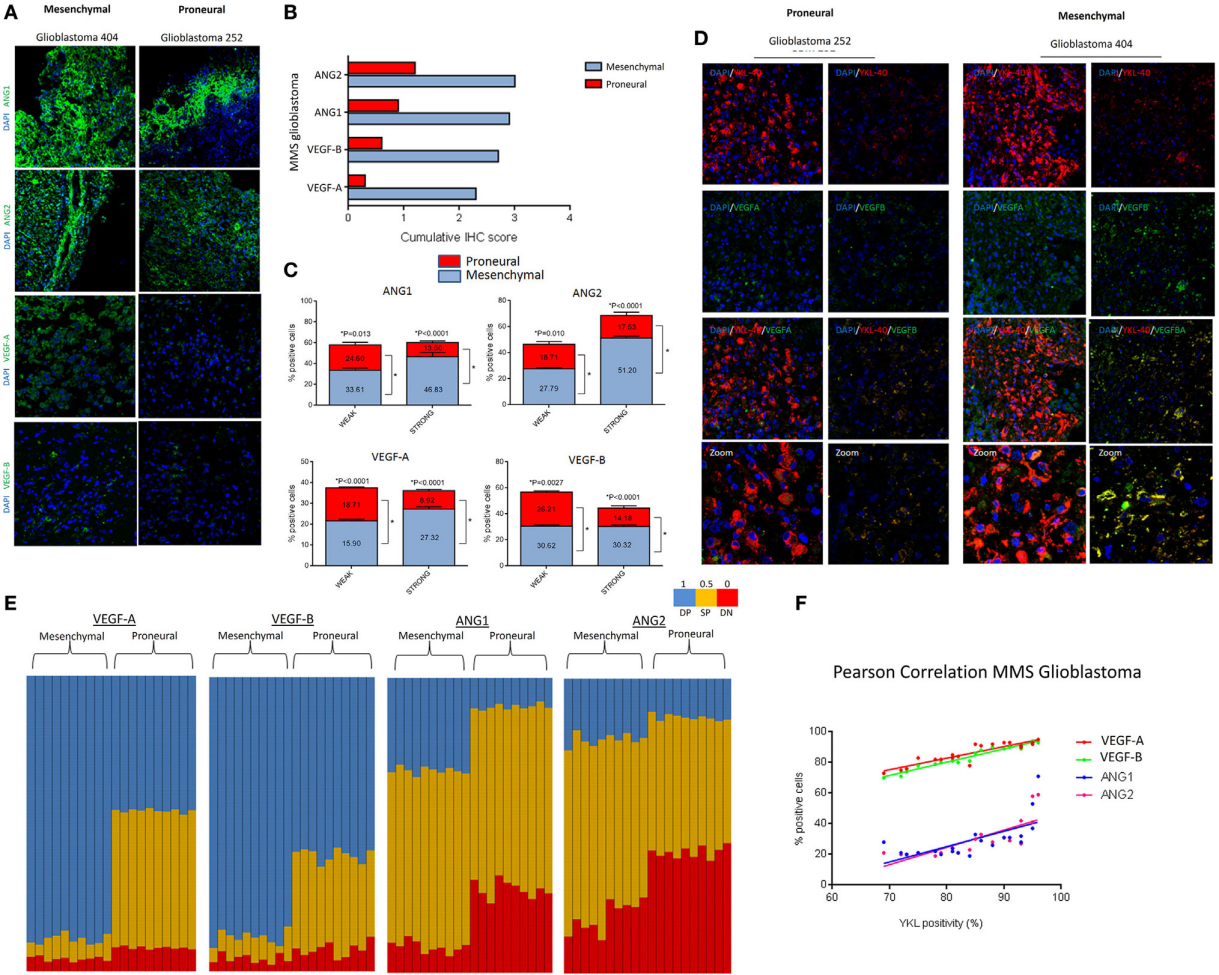
Minimal multigene signature (MMS) glioblastoma effectively segregates preclassified tumors. **(A)** Immunostaining of MMS glioblastoma genes in representative glioblastoma tumors. **(B)** Derivation of immunohistochemistry score for each of the MMS glioblastoma genes in glioblastoma tumors. **(C)** Semiquantitative analysis of individual cancer cells differentially expressing each of the specific MMS glioblastoma genes. **(D)** Coexpression of YKL-40 and vascular endothelial growth factor A (VEGF-A), vascular endothelial growth factor B (VEGF-B) in representative glioblastoma tumors. **(E)** Single-cell profiling for coexpression of YKL-40 and each of the MMS glioblastoma genes. 1,000 tumor cells were plotted for both proneural and mesenchymal glioblastoma tumors (*n* = 10 each subtype). **(F)** Pearson correlation analysis of individual MMS glioblastoma proteins and YKL-40 in tumor regions. Individual MMS glioblastoma genes are positively correlated with YKL-40 expression (VEGF-A, *r* = 0.9042; VEGF-B, *r* = 0.9751; angiopoietin 1 (ANG1), *r* = 0.6596; and angiopoietin 2 (ANG2), *r* = 0.7850).

Multigene signatures to predict patient prognosis in glioblastoma involve a complex strategy, computational models, or sequencing-based mutation identification (35–37). To analyze whether MMS glioblastoma can segregate proneural and mesenchymal tumors histopathologically, we performed IHC of MMS glioblastoma of 24 preclassified glioblastoma tumors (*n* = 12 of each proneural and mesenchymal glioblastoma). Bright, uniformly stained, tumor regions were evident in preclassified mesenchymal glioblastoma tumors, but not in proneural glioblastoma tumors that showed patched and/or weaker staining of MMS glioblastoma genes (Figure 5A). Furthermore, intensity scoring of the immunohistochemical staining (15) on a scale of negative (0), weak (1), medium (2), and strong (3) revealed higher cumulative scores of MMS glioblastoma in the mesenchymal glioblastoma tumors as compared to the proneural subtype (ANG1, 2.9 vs 0.9; ANG2, 3.0 vs 1.2; VEGF-A, 2.3 vs 0.3; and VEGF-B, 2.7 vs 0.6) (Figure 5B). The mesenchymal subtype scored either 2+ or 3+ for MMS glioblastoma and the proneural subtype showed only weak positivity (Figure S2 in Supplementary Material). Since MMS glioblastoma genes are angiogenesis regulators, we excluded physiological regions of hypoxic with the high level of angiogenesis and necrosis to avoid bias in our analysis. We extended our study to the single-cell level by scoring individual cancer cells. For this purpose, brightly stained cancer cells within each field were considered as strongly positive, and a normalized intensity value was calculated using Image J. A weakly positive cell had a <5-fold normalized intensity value of a strongly positive cell. Semiquantitative measurements of MMS glioblastoma (*n* = 10 for each subtype; >1,000 cells/glioblastoma) showed a higher percentage of strongly positive cancer cells in mesenchymal subtype as compared to the proneural subtype (ANG1, 46.8 vs 13.5, ***p < 0.001; ANG2, 51.2 vs 17.5, ***p < 0.001; VEGF-A, 27.3 vs 8.9, **p < 0.001; and VEGF-B, 30.3 vs 14.2, ***p < 0.001) (Figure 5C). Similarly, percentages of weakly positive cells were higher in the mesenchymal subtype than in the proneural subtype (ANG1, 33.6 vs 24.6, *p < 0.01; ANG2, 27.8 vs 18.7, **p < 0.001; VEGF-A, 21.7 vs 15.9, ***p < 0.001; and VEGF-B, 30.6 vs 26.2, ***p < 0.001) (Figure 5C). Normally, YKL-40 expression is associated with hypoxic and invasive regions of tumors, and therefore, YKL-40 is a widely used mesenchymal subtype marker (25, 38–40). However, YKL-40 expression was not limited to these regions in our analysis. Next, we analyzed whether YKL-40 positivity correlated with expression of MMS glioblastoma genes. Both, subtype tumors with strong YKL-40 positivity coexpressed VEGF-A, VEGF-B (Figure 5D) and ANG1, ANG2 (Figure S4 in Supplementary Material). Moreover, we scored individual cancer cells in multiple regions of the glioblastoma tumors (*n* = 1,000 cells/tumor; *n* = 10 of each glioblastoma subtype) and generated coexpression maps of YKL-40 and VEGF/ANG. Cancer cells were scored as either SP, DP, or DN for the respective angiogenesis-related proteins. Particularly, the mesenchymal subtype consisted of >80% cancer cells that were stained for both YKL-40 and VEGF-A or VEGF B, whereas the proneural subtypes showed >60% (VEGF-A) and <40% (VEGF-B) cancer cells that coexpressed these markers (Figure 5E, blue columns). We also found a significant fraction of single SP cells for VEGF-A or VEGF-B, respectively, that were present specifically in the proneural glioblastoma subtype (Figure 5E, green columns). Immunohistochemical staining of both YKL-40 and ANG coexpression in both subtypes showed more SP cells (green columns) than cells coexpressing both YKL-40 and ANG (Figure 5E). Furthermore, Pearson correlation analysis of MMS glioblastoma with YKL-40 expression showed a positive correlation with YKL-40 expression (Figure 5F). Particularly, VEGF-A and VEGF-B showed a stronger positive correlation (VEGF-A, *r* = 0.9042, ****p < 0.0001; VEGF-B, *r* = 0.9751, ****p < 0.0001) with ANG (ANG1, *r* = 0.6596, **p < 0.0001; ANG2, *r* = 0.7850, ****p < 0.0001) (Figure 5F; Figure S3 in Supplementary Material). Furthermore, the large YKL-40-negative tumor regions did not express MMS glioblastoma genes (Figures S5 and S6 in Supplementary Material). Thus, our study provides evidence that MMS glioblastoma genes can mark mesenchymal glioblastoma cells both *in vitro* and *in vivo*.

### MMS Negatively Correlates with Glioblastoma Patient Survival

**Figure 6:**
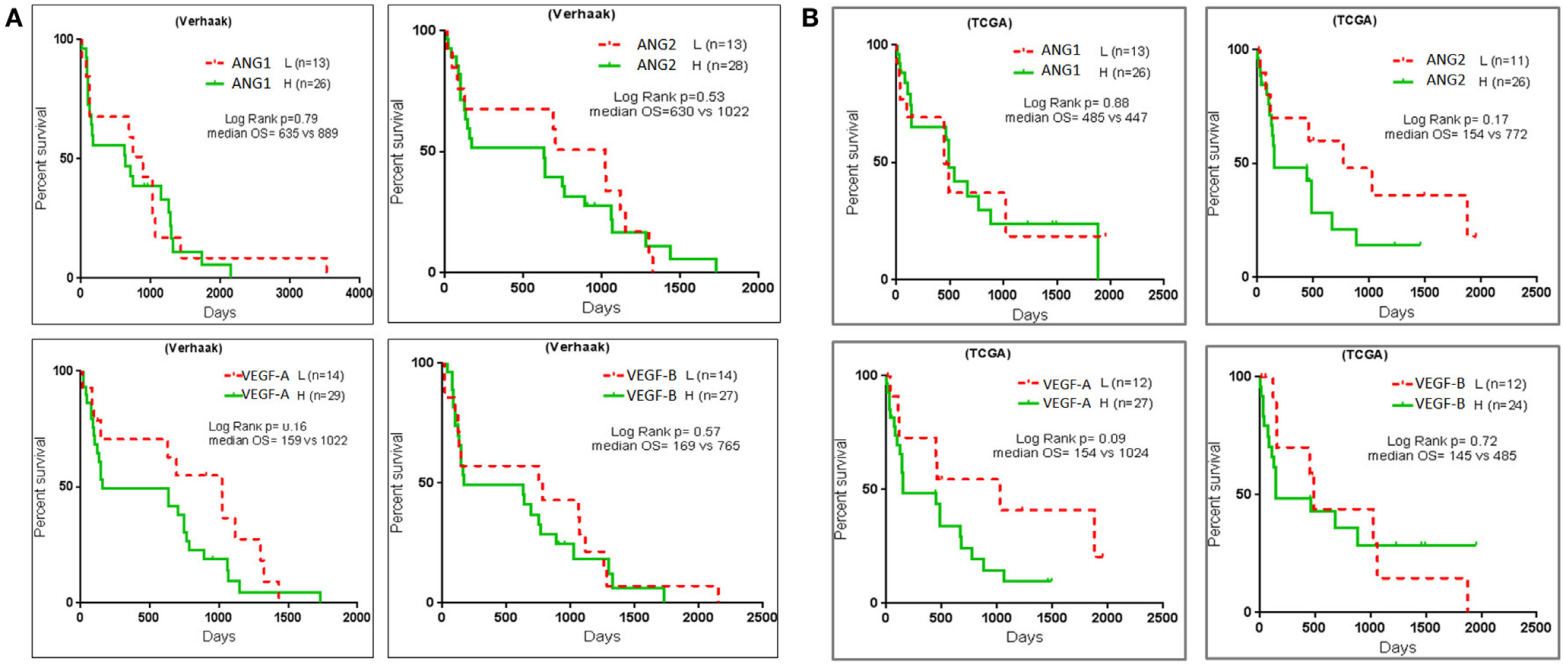
Multigene signature predicts survival of glioblastoma patients. Kaplan–Meir survival curves with the use of glioblastoma data sets **(A)** Verhaak data set for glioblastoma patients survival with each of the multigene signature ANGPT1, ANGPT2, vascular endothelial growth factor A (VEGF-A), and vascular endothelial growth factor B (VEGF-B). **(B)** Patient survival prediction was calculated on the basis of TCGA glioblastoma data set. Patients in both the data sets were segregated into classes with low and high expression for each of the MMS glioblastoma genes, respectively.

We performed survival analysis in two large glioblastoma data sets—Verhaak and TCGA (5, 6), to determine prognostic significance of MMS to discriminate the proneural mesenchymal glioblastoma subtypes. For each data set, we segregated patients into STSs and LTSs and acquired two clinical subgroups (17). Survival plots showed that high expression of MMS glioblastoma resulted in poor patient survival in both the data sets (Figures 6A,B). Furthermore, we observed that patients with low expression of all four MMS glioblastoma genes ANG1, ANG2, VEGF-A, and VEGF-B survived longer than patient with high expression of angiogenesis genes. The low *p* values in survival curves may be caused by the presence of regions of heterogeneity in patients’ tumor tissues.

Our data strongly emphasize that glioblastoma tumors can be successfully categorized into the two major subtypes on the basis of expression of the genes ANG1, ANG2, VEGF-A, and VEGF-B. This subclassification can become useful in the design of personalized therapy of glioblastoma patients.

## Discussion

Various higher-grade glioma cell cultures have been established by us and we report here the development of two stable prototype cultures KW10 and MTA10 that represent proneural and mesenchymal subtypes of glioblastoma, respectively. KW10 cells showed expression of stemness genes, formed neurospheres, and more importantly made highly infiltrative tumors, all features representative of the mesenchymal phenotype.

The two clinically interrelated glioblastoma subtypes proneural and mesenchymal can undergo proneural to mesenchymal transition often in response to therapy (9, 10, 41). Mesenchymal glioblastoma is the most aggressive subtype with high expression of the four angiogenic genes, which is therapy refractory and highly invasive (25, 40, 42). In highly vascularized tumors, complex interplay of VEGFs and ANGs is known to regulate angiogenesis by supporting endothelial cell growth and stabilizing vessels (43, 44). However, it was not known whether mesenchymal cancer cells also coexpress angiogenesis-related genes such as VEGFs and ANGs. Therefore, the well-vascularized nature of glioblastoma led us to determine whether the two subtypes differ in expression of genes involved in angiogenesis. An in-depth investigation of the two cell cultures revealed that MMS of the four genes ANG1, ANG2, VEGF-A, and VEGF-B enabled proneural and mesenchymal glioblastoma subtype identification. On the other hand, VEGF is also known to attenuate migratory potential of cancer cells and thereby decrease their mesenchymal nature (39).

In this study, we categorized glioblastoma tumors on the basis of expression of YKL-40 and Olig2 using a novel algorithm for semiquantitative scoring of “tumor regions” that were stained for each of these markers. YKL-40-positive tumors were segregated into two subgroups either with intense and uniform staining or with low, restricted expression. Similarly, OLIG2-stained tumors showed either strongly OLIG2-positive areas within the major part of the tumors or weakly low Olig2 positive areas with restricted staining. Hence, we designed a histopathology-based approach to map differential YKL-40 and OLIG2 expression patterns and generated a semiquantitative score for subtyping. By analyzing regions of YKL-40/OLIG2 expression, we preclassified glioblastoma tumors into proneural and mesenchymal glioblastoma subtypes (35/38; segregation efficacy 92.0%). Our data elaborate an important fact that YKL-40^high^/OLIG2^low^ tumors belong to the mesenchymal subtype, whereas YKL-40^low^/OLIG2^high^ tumors belong to the proneural subtypes. Hybrid cancer cells coexpressing multiple subtype markers have been found in glioblastoma (33). We found in our study in preclassified glioblastoma tumors and cells resides in ROH coexpressed both YKL-40 and OLIG2 proteins. We hypothesize that these “hybrid tumor regions” necessitate examination of larger tumor areas before any subtype-specific information can be retrieved from histopathology. As far as we know, we report here the existence of distinct regions in glioblastoma tumors. Recently, ROH regions (on the basis of metabolism, invasive regions, and angiogenic regions) were reported for glioblastoma (45).

Minimal multigene signature glioblastoma was applied to preclassified glioblastoma tumors to evaluate its subtyping potential. Together, higher IHC scores for each of the MMS glioblastoma genes obtained by multiregion analysis proved segregation efficiency of our gene set. Collectively, we show that MMS glioblastoma can effectively label mesenchymal glioblastoma cancer cells both *in vitro* and *in vivo*. Furthermore, we investigated coexpression of MMS glioblastoma genes and the YKL-40 gene. Hypoxia is a known driver of YKL-40 expression in glioblastoma (25). Strikingly, our data revealed coexpression of MMS glioblastoma molecules with YKL-40 in both subtypes. Therefore, we hypothesize that another regulation mechanism of YKL-40 expression occurs in glioblastoma. To extend our findings from single cell to a tumor, we screened 1,000 cells/tumor and categorized individual cells into SP (one gene of MMS), DP (one of MMS glioblastoma gene and YKL-40), or DN (not expressing any of the MMS glioblastoma genes and YKL-40). Indeed, individual cancer cell coexpression analysis showed strong colocalization of YKL-40 and MMS glioblastoma proteins. Statistically, we found a higher correlation between the expression of YKL-40 and VEGFs (VEGF-A and VEGF-B) as compared to ANG1 and ANG2. Our data indicate that expression of VEGF-A and VEGF-B is valuable for segregation of the two glioblastoma subclasses and are thus a good alternative for YKL-40. Spatiotemporal intratumoral ROH in glioblastoma is a major limitation of the translation of molecular subtypes knowledge into the clinic. For example, collective use of multiple marker sets for validation of the cellular phenotype is not practical during histopathological examination and can cause complexity in the analysis (9, 38, 46). In conclusion, our minimal gene signature enabled us to successfully categorize 83% of preclassified glioblastoma.

Our data elucidate that larger areas of tumor are necessary for accurate histology based on proneural and mesenchymal subtyping. In conclusion, our study demonstrates a novel histology-based gene signature that effectively segregates proneural and mesenchymal subtype tumors, which may have tremendous ramifications for personalized medicine.

## Ethics Statement

Study on human glioblastoma patient samples was approved by the Institutional Ethics Committee (IEC), Seth GS Medical College & KEM Hospital, Mumbai (Reference no: EC/GOVT-2/2012), and IC-SCR committee of National Centre for Cell Science (NCCS), Pune, India. Use of animals for this study was approved by Institutional Animal Ethics Committee (IAEC) (Reference No. EAF/2011/B-169), NCCS, Pune, India.

## Author Contributions

AShiras: conceptualization of work, data analyses, and manuscript writing. ASharma: experimental work, data analyses, and preparation of manuscript draft. AB: experimental work, preparation of figures, and data analyses. AM: experimental work and preparation of figures; DM: help in providing clinical tumor tissue samples and data analyses. NG: histopathological analyses and clinical grading of tumor tissues.

## Conflict of Interest Statement

The authors declare that the research was conducted in the absence of any commercial or financial relationships that could be construed as a potential conflict of interest.

## Abbreviations

ANG1, angiopoietin 1; ANG2, angiopoietin 2; VEGF-A, vascular endothelial growth factor A; VEGF-B, vascular endothelial growth factor B; YKL-40/CHI3L1, chitinase-3-like protein 1; FN1, fibronectin; VIM, vimentin.
