# Supplementary figures and images for: Angiogenic Gene Signature Derived from Subtype Specific Cell Models Segregate Proneural and Mesenchymal Glioblastoma
Source: Front Oncol. 2017 Jul 11;7:146. doi: 10.3389/fonc.2017.00146 (PMC5504164; doi:10.3389/fonc.2017.00146)

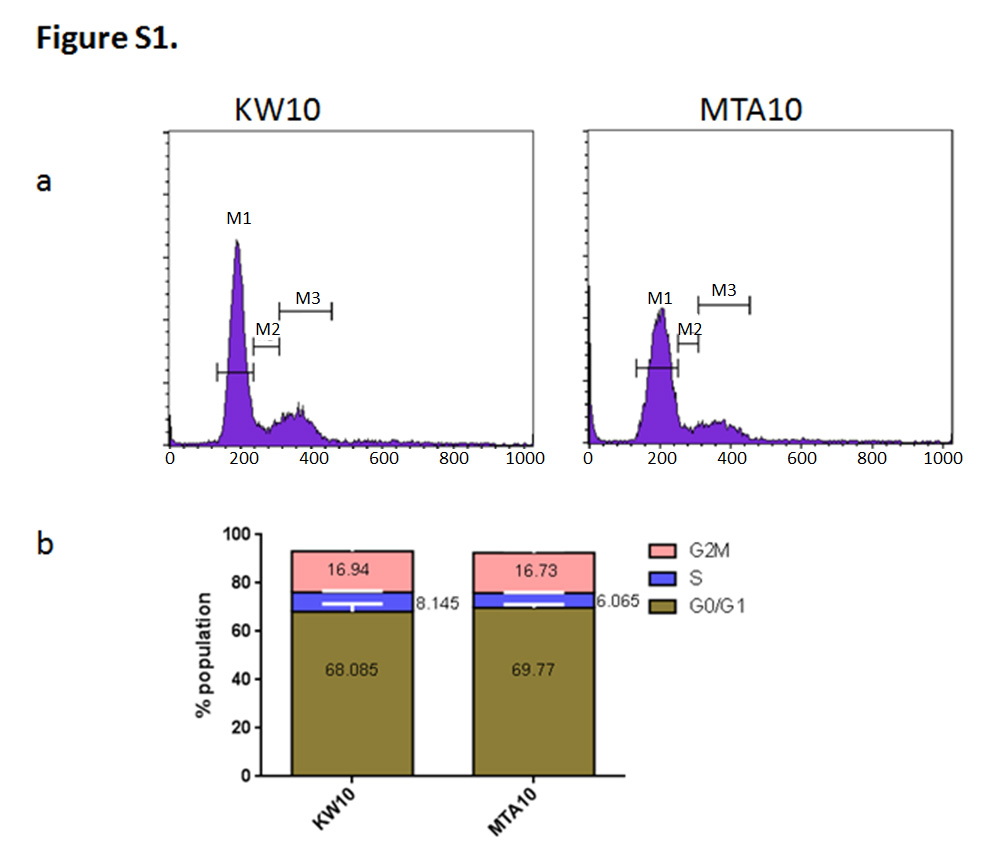

Supplement: Supplementary file 2 [file Image_1.jpg]

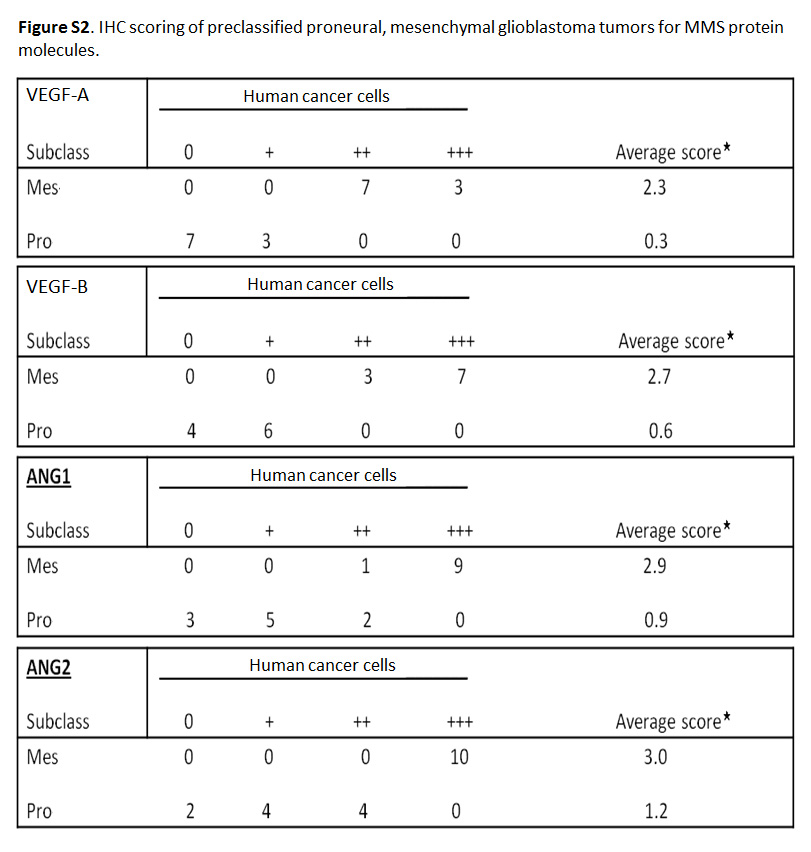

Supplement: Supplementary file 3 [file Image_2.JPEG]

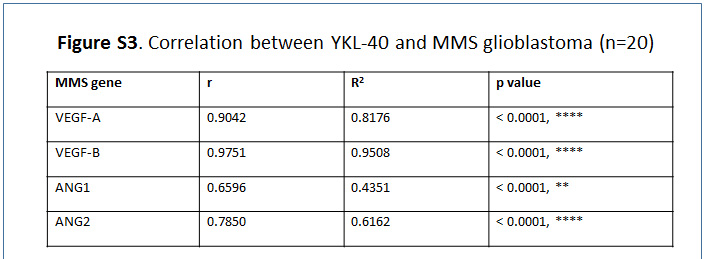

Supplement: Supplementary file 4 [file Image_3.JPEG]

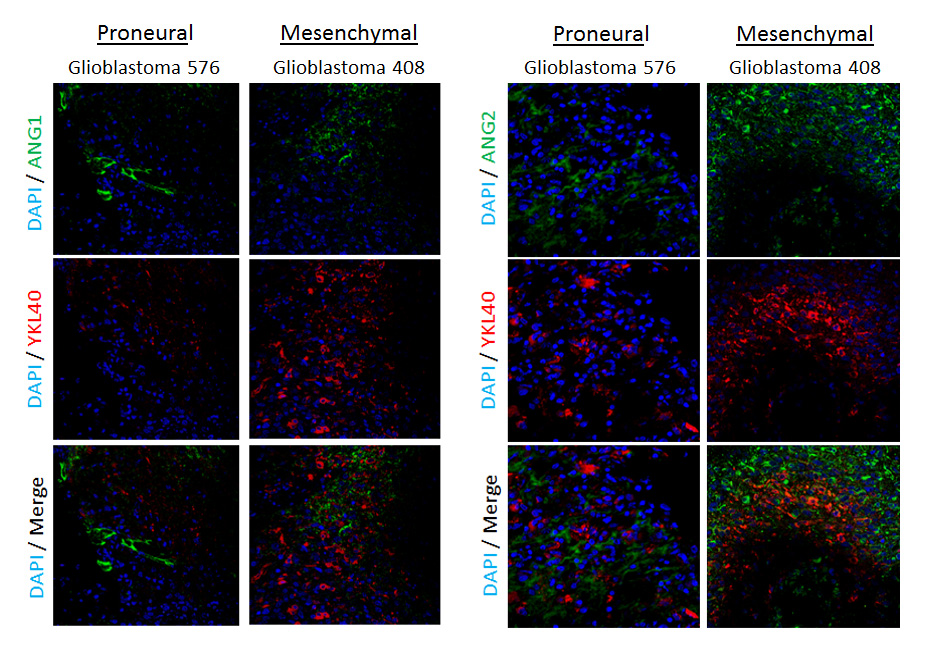

Supplement: Supplementary file 5 [file Image_4.JPEG]

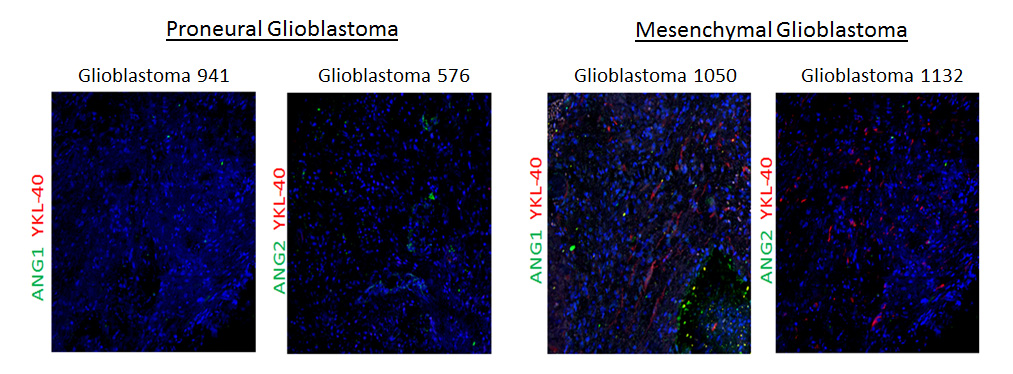

Supplement: Supplementary file 6 [file Image_5.JPEG]

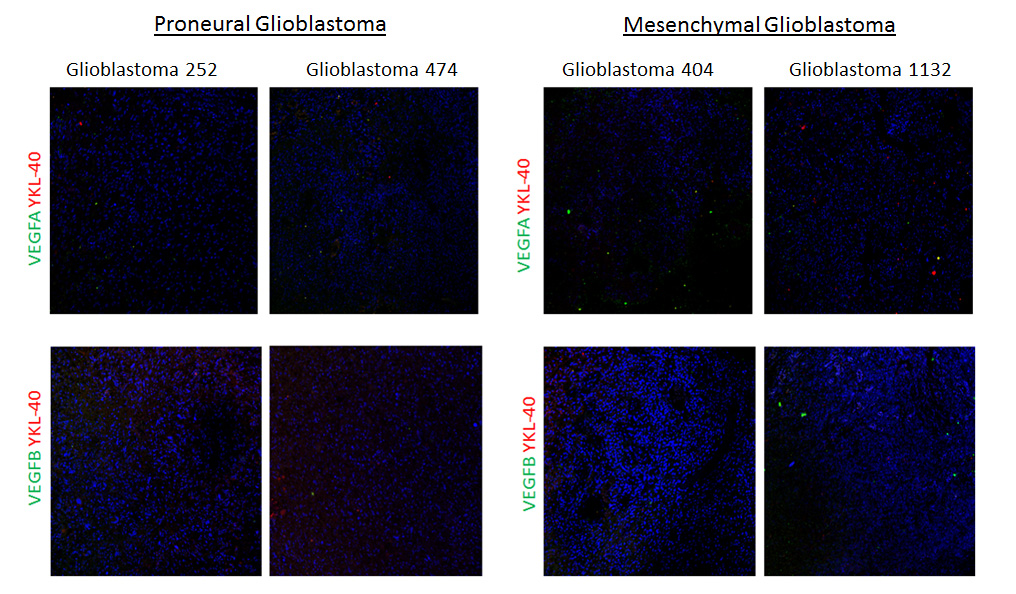

Supplement: Supplementary file 7 [file Image_6.JPEG]
